# Identification and annotation of milk associated genes from milk somatic cells using expression and RNA-seq data

**DOI:** 10.6026/97320630018703

**Published:** 2022-08-31

**Authors:** Neelam Krishna, Shraddha Vishwakarma, Pramod Katara

**Affiliations:** 1 Computational Omics Lab, Centre of Bioinformatics, University of Allahabad, Prayagraj - 211002, India

**Keywords:** Casein, Somatic cell, Lactation, Milk Traits, Gene expression

## Abstract

It is of interest to identify and annotate milk associated genes using expression profiling and RNA-Seq data from milk somatic cells. RNA-Seq data was pre-processed and mapping was done to identify differentially expressed genes (DEG). The functional insights about the up and down regulated genes were gleaned using the protein-protein interaction Network in the STRING database followed by CytoHubba analysis in Cytoscope. Gene ontology, annotation and pathway enrichment was completed using ShinyGO, David tool and QTL analysis. These analysis shows that 21 genes are linked with the secretion of milk.

## Background:

Cow milk represents important nutritional support to human health; in particular, it is a rich source of all nutrients such as protein, fat, and minerals that are required for human growth. Studies based on clinical evidence also support the consumption of cow milk and dairy products for better health; it is also reported to be beneficial for cardio metabolic health [1]. Milk composition has a dynamic nature which comprises immunoglobulins, hormones, growth factors, cytokines, nucleotides, peptides, polyamines, enzymes, and other bioactive peptides [2]. The composition and quality of milk vary with the stage of lactation, age, breed, nutrition, and health status of the udder. The health of the udder is one of the major factors which affect the quality of raw milk [3]. Milk Somatic cells (SCs) in a cow are a mixture of milk-producing cells and immune cells, i.e., WBC (White blood cells), they are associated with udder health and milk production. It is a known process that somatic cells are released into the cow milk to defend against udder infections, i.e., mastitis, these cells fight with infection and also repair the tissue damage. Milk SC is influenced by various factors including cow productivity, health, parity, lactation stage, and breed [4]. Change in any kind of environmental conditions and increased stress significantly increases the amount of SC in milk. In developed countries, milk somatic cell counts (SCCs) are in practice as a marker, mainly to monitor the udder-infection and as an indicator of raw milk quality [5]. Somatic cells are also in practice to measure the shelf-life of pasteurized fluid milk. It is now well established that there is a direct correlation between the number of somatic cells and udder infection, which is directly associated with milk quality. There is a need to observe the expression profiles of the genes belonging to somatic cells of the udder, which allows understanding of the involvement of genes and their regulation process, and their associations with milk quality and production rate [6]. Milk protein and fats are major constituents of milk and these traits are genetically influenced by associated genes. The use of expression profiling and genome wide gene expression is common now a day's [7], dairy science also reported studies based on expression profiling [8]. Therefore, the analysis of expression patterns of genes of interest is a key step to understanding the production and quality rate. During the lactation period expression of milk somatic cells may help to identify more essential genes that are involved in milk production and in its formation. Mammary transcriptome in dairy cows revolved around the cattle's health and milk production [9]. Availability of gene expression data, i.e., microarray and RNA-seq, and Bioinformatics resources for functional genomics make it feasible to perform functional genomics in a more advanced and precise manner [10]. Considering the facts current research aims to find out genes that affect the milk production and composition during the early and late days of lactation, for that differential expression of the gene and network analysis has been done. The expression level of genes will facilitate understanding of particular genes that enhance milk production and genes that decline milk production. QTLdb is used to identify genes that are directly involved in milk production and composition traits [11, 12]. Therefore, it is of interest to identify and annotate milk associated genes using expression profiling and RNA-Seq data from milk somatic cells.

## Material and methodology:

### Data collections:

To perform the expression profiling RNA-seq data of Holstein cows were retrieved from the GEO database (GSE60575) [13]. It includes 21 biological samples (SRP045641; Bio project ID PRJNA258561) and is extracted from somatic cells belonging to Holstein bovine milk [14]. This data contains 101 SRA runs which are paired-end layout reads. For the analysis purpose, data has been divided into 2 different groups: High yield and low yield, based on their lactation periods from early days of lactation to late days of lactation.

### Quality control and Quantification:

FASTX Toolkit was used to check the raw reads quality and pre-processing. Raw reads were filtered based on the Phred score Q33 by the FASTQ quality filter and end-trimming raw reads based on the quality by FASTQ Quality Trimmer [15]. Pre-processed data was used for quantifying transcripts abundance from RNA-Seq data using the Kallisto program [16].

### Differentially expressed genes and Network analysis:

Analysis of differentially expressed genes between two conditions, i.e. high yield of milk and low yield of milk, using p-value < 0.05 and |log2fc| > 2 by Sleuth was performed. Fold change is estimated and overall expression strength is indicated by the identification of the coefficient [17]. Further, network analysis of identified DEGs was performed by STRING (v11.5) [18] and Cytoscape [19]. (see PDF) [20.

### Gene enrichment and Pathways analysis:

Highly interacted top-ranked genes obtained from Cytohubba were used for gene ontology (GO) analysis which was carried out with ShinyGO [21] and functional annotation tool DAVID [22] with P-value cut-off 0.05. Further for QTL analysis, differentially expressed genes were mapped with QTLdb [23]. Identification of QTL genes was observed and their involvement was checked in milk production and composition traits (Figure 1 - see PDF).

## Results and Discussion:

### RNAseq data and differentially expressed gene:

Collected raw reads from 21 SRA experiments were pre-processed with FASTX, and then mapped with Bos_taurus_UMD_3.1.1 genome by pseudo alignment tool, Kallisto. Moreover Bos_taurus_UMD_3.1.1 reference assembly was aligned approximately up to 85.5% and quantified in total 76,341 transcripts identified from Kallisto. Further data was normalized and resulted in 14,433 differential expressed genes by setting up a threshold p-value < 0.05 between high yielding and low yielding milk production. Out of these genes, 1231 elevated expression of genes > +.2 FC (fold change) and 360 down-regulated genes < -2 FC were identified by setting up threshold values for |log2fc| > 2.

### PPIs Network:

The Protein-Protein Interaction Network provides the connection between proteins which helps us to understand the role of proteins in a systemic manner. Predicted up (1231) and down-regulated (360) genes were used to construct the PPI networks. PPI network has been observed through a string database [24], separately for upregulated and down-regulated genes and network characteristics of both the network have been examined (Table 1 - see PDF).

For further analysis, these PPI networks have been imported to a network analyzer and visualization tool, i.e., Cytoscape. Cytohubba plugin has been used to identify the most interactive, top-ranked 50 nodes for upregulated and down-regulated PPIs networks (Table 4 - see PDF). Upregulated most interactive, top-ranked nodes are represented by blue color while pink color nodes are portrayed with top-ranked 50 down-regulated genes (Figure 2 - see PDF). The molecular network displayed that these proteins have high connectivity among them and make a network motif. The network also suggested that top-ranked genes consist of more casein protein-related genes; Caseins protein is a major constituent of milk, which is highly interacted [25].

In Figure 2(see PDF), yellow nodes (shown in the blue and pink colour top most ranked 50 genes upregulated and down-regulated PPI networks) have been identified as the QTL gene that is mapped by the QTLdb database for milk yield, milk protein, and milk fat traits in dairy cattle. In the network, these QTL genes are well connected to each other. The genes, i.e., LTF, GLYCAM1, CSN1S1, CSN1S2, CSN2, CSN3, PIGR, and PAEP are made compact structures altogether and singly connected to the rest of the blue color network and signified as milk trait module by analyzing the network [26]. Eight genes, i.e., GNAS, PTPN11, SELL, STAT1, STAT6, STAT5B IL10RA, and CD44 are shown as yellow color nodes among pink color nodes network (Figure 2b - see PDF) for down-regulated top most 50 gene PPI network. These nodes well interact in a circular module of a network; it's not made a compact structure while it is part of a well compact, circular structure of the network. In both networks, yellow color nodes are QTL genes that are signified as milk production and composition traits associated genes. All top ranked genes are further used for annotation and pathways enrichment analysis based on fold change enrichment.

### Functional annotations and pathway enrichment:

To find out the additional information regarding predicted up and down-regulated genes and their functional annotation and pathway enrichment were performed. Annotation results suggest that upregulated genes, i.e., PRPF39, HNRNPUL1, RBM39, HNRNPA2B1, SRRM1, HNRNPD, RPL35A, SRSF2, and PCBP2 are mainly involved in the necessary function, such as RNA processing, RNA metabolism, and Ribonucleoprotein complex. CSN3 prevents casein precipitation in milk; LALBA activates LS (Lactose synthase) to synthesize lactose and forms a major carbohydrate component of milk [27,28]. PAEP act as the Primary component of whey protein, and LTF transferrins are iron binding transport proteins and are associated with the binding of an anion. Other three genes, namely, CSN1S1, CSN1S2, and CALB1 play crucial roles in the capacity of milk to transport calcium phosphate and calcium-binding [29,30].

Down-regulated genes such as CASC3, POU2AF1, STAT6, EIF3CL, ADAR, STAT1, SMG5, PDCD11, STAT5B, and BCL6 control the innate immune response, Macromolecule metabolic process, and Cytokine-mediated signalling pathways during late days of lactation [31]. The genes, i.e., RPS27L, RPL34, RPL7, RPL8, RPL6, RPS24, and SAFB are involved in major biological Reactome pathways such as Metabolism of proteins, Translation, and Metabolism of RNA. Nonsense Mediated Decay (NMD) independent of the Exon Junction Complex (EJC) pathways maintains the expression of the genes and reduces the error in gene expression [32]. Down-regulated genes, i.e., PTPN11, STAT6, CSF2RB, and STAT1 are involved in 3 Reactome pathways (Table 2 - see PDF), which mainly belong to the immune system signalling process [33, 34].

### Identification of genes associated with milk production and composition:

In total, 15 candidate functional genes have been identified, which are known to affect milk production and composition traits in which 7 up-regulated genes and 8 down-regulated genes are associated with the total 63 traits of milk quality and quantity. Comparative analysis with previously reported genetic data including QTL mapping was used for the assessment of the position of DEG's. The physical position of each DEG with the position of known QTLs that have been shown to be associated with the milk yield, milk protein, and milk fat traits in dairy cattle from the QTLdb database has been examined [35]. Through QTL mapping it has been observed that in total 67,849 QTL are involved in milk, out of the 5,532 QTL are involved in milk production, 39,536 QTL are involved in milk composition fat, 20,108 QTL belongs to milk composition protein, and 2,673 belong to other milk nutrients [36, 37]. Highly expressed and interacted genes are also associated with the protein and fat composition of milk, which are influenced by nutrition, stage of lactation, and breed of cattle [38], GLYCAM1 and CSN2 play a major role in the milk fat composition and CSN1S1, CSN1S2, CSN2, CSN3 are involved in protein composition [39]. LTF is mainly involved in the composition of Milk lactose content, Milk urea nitrogen content, and Milk alpha-S1-casein percentage [40]. Milk solids percentage and milk somatic counts are influenced by the expression of PAEP gene (Table 3 - see PDF), that is well reported in previous studies [41].

In down-regulated gene group, genes such as GNAS and PTPN11 are reported to be important for oocyte maturation and embryo development, and also act as growth factors [42]. SELL gene involved in mastitis, biological process reported [43]. STAT1 and STAT6 are involved in calculating yield grade and maintaining cow body weight and health and STAT5B have an almost fatalistic attitude towards milk fat and protein composition, which directly affect milk quality during the late days of lactation [44]. Negative expression of IL10RA protects the cow from M. paratuberculosis susceptibility and it is also reducing the number of somatic milk cells which directly affects the milk production rate [45]. The presence of CD44 affects lipid metabolism and lactation persistence [46]. Available reports justify that the expression level of upregulated genes helps to enhance the milk production and composition rate in the early days of lactation and down-regulated genes degrade the milk quality and quantity in the late days of lactation [47].

## Conclusion:

Analysis shows that some genes are highly upregulated and are linked with milk yield and its composition. Data shows that 6 genes namely RPS27L, RPL34, RPL7, RPL8, RPL6 and RPS24 are found to be involved in the secretion of milk proteins. Data further shows that 9 down-regulated genes (PTPN11, CD44, GNAS, IL10RA, SELL, STAT5B, STAT1, STAT6, and PAEP) are linked to affect milk yield and its composition.
